# Comparative Clinical Evaluation of Two Techniques in the Arthroscopic Treatment of Partial Articular Rotator Cuff Tears after Six Years of Follow-up

**DOI:** 10.1055/s-0044-1790211

**Published:** 2024-12-07

**Authors:** Guilherme do Val Sella, Luciana Andrade da Silva, Ricardo Makoto Okamoto, Hector Carmona Marmille, Pedro Gabriel Pelegrino do Val, Alberto Naoki Miyazaki

**Affiliations:** 1 Grupo de Cirurgia de Ombro, Departamento de Ortopedia e Traumatologia, Faculdade de Ciências Médicas, Santa Casa de São Paulo, Pavilhão Fernandinho Simonsen, São Paulo, SP, Brasil

**Keywords:** arthroscopy, rotator cuff, treatment

## Abstract

**Objective** To clinically evaluate the medium-term results of the arthroscopic treatment of partial-thickness rotator cuff tears (PTRCT) using the transtendon repair (TTR) technique and the tear completion repair (TCR) technique through the modified University of California, Los Angeles (UCLA) Shoulder Rating Scale, the Constant-Murley score, and force analysis.

**Methods** The present was a retrospective reevaluation study of cases operated on arthroscopically for PTRCT after a minimum follow-up of 6 years. There were 34 patients, 18 of whom underwent TTR and 16, TCR. We compared the current UCLA and Constant-Murley scores, the mean strength between the techniques, and the UCLA score currently and 2 years after surgery for the same group, as published in a previous study, to assess whether or not the results changed throughout time.

**Results** There was no statistical difference regarding the scores. The current UCLA scores were of 33.8 for the TTR and of 32.9 for the TCR ( *p* = 0.113), and the current Constant-Murley scores were of 91.9 and 86.8 respectively ( *p* = 0.075). For the TTR, the previous UCLA score was (2 years postoperatively) of 32.4 and the current score was of 33.8 ( *p* = 0.374); for the TCR, the score after 2 years was of 32.4, and the current score was of 32.9 ( *p* = 0.859). In the TTR, the mean strength was statistically higher on the dominant side (11 kg) than on the non-dominant side (7.80 kg) ( *p* = 0.023) and those of the TCR (8.25 kg) ( *p* = 0.042).

**Conclusion** There was no statistical difference in the medium term when comparing the UCLA and Constant-Murley scores concerning the technique used (TTR or TCR), nor was there any change in UCLA scores between 2 and 6 years postoperatively. Among the patients submitted to TTR, the mean strength was statistically higher on the dominant side than on the non-dominant side, and higher than that of the patients submitted to TCR.

## Introduction

Partial rotator cuff injury is a major cause of shoulder dysfunction and pain. 1 Its incidence is not yet a consensus in the literature, although there are studies 2 3 4 showing values of 14.5 4 to 32%. 3 Fukuda also describes that around 1/3 (27%) of these lesions affect the joint portion. 4

Ellman was the first to propose, based on arthroscopic findings, a system to classify partial lesions in terms of their location (A: articular; B: bursal; C: intratendinous) and their extent (grade 1: < 3 mm; grade 2: 3–6 mm; grade 3: > 6 mm). 5

The pathophysiology of partial-thickness rotator cuff tears (PTRCT) is believed to involve both intrinsic (areas of hypovascularization and age-related metabolic alterations) and extrinsic mechanisms (internal superior posterior impingement, acute traumatic events, and repetitive microtraumas), or even a combination of these. 6 7

The initial treatment of PTRCTs is carried out with conservative measures, such as physiotherapy and changing habits to prevent the progression of the lesion 8 . Surgical treatment is indicated in the failure of this or in cases in which the lesion affects more than 50% of the tendon thickness due to a risk of up to 40% in its progression. 1 9

Different arthroscopic surgical techniques have been described to approach this type of injury, such as simple arthroscopic debridement of the tendon, 2 6 10 11 12 or tendon repair. The repair techniques used are the TTR, initially described by Snyder, 13 or the tear completion repair (TCR). 8 11 14 15 16 17 18 19 There are studies with short-term evaluations comparing which surgical technique would be the best to treat PTRCT clinically, 8 16 17 18 20 21 22 and all of them concluded that there is no statistical difference between them. A few works 1 23 24 25 carry out this comparison in the long and medium terms. The aim of the present work is to reevaluate patients operated on through both of the aforementioned techniques, whose results were previously published, 21 now with medium-term follow-up and with analysis of the current strength to assess whether there has been a change in the results.

## Materials and Methods

From October 1999 to December 2016, 39 patients diagnosed with PTRCT underwent arthroscopic surgical treatment by our institution's shoulder group. All operated cases were classified as Ellman 3 (partial lesion greater than 50% of tendon thickness). None of the cases had been submitted only to surgical debridement, and repair of the lesion was performed in all cases. Two arthroscopic repair techniques were used: TTR on 19 patients (group I), and postinjury repair (TCR) on 20 patients (group II). The choice of surgical technique was at the surgeon's discretion, and the evaluation was retrospective. Have been clinically evaluated and compared in a previous study 21 after a minimum of 2 years of follow-up.

For the current study, 18 patients (94.7% of the initial group) operated on through the TTR technique (group III) and 16 (80% of the initial group) submitted to the TCR technique (group IV) were contacted.

Group III had a mean follow-up of 105.6 months (8.8 years; range: 6–15 years); 12 patients were male (66.7%), and 6 were female. The mean age was of 52.2 (range: 38–74) years. A total of 72.2% of the cases affected the dominant side (DS), and 15 patients (83.3%) practiced sports. In nine cases, there was history of trauma (in these cases, acromioplasty had not been performed), and in six cases, there was a diagnosis of superior labrum, anterior to posterior (SLAP) injury, for which tenotomy and tenodesis were performed in five cases, and in one case, debridement.

Group IV had a mean follow-up of 91.2 months (7.6 years; range: 6–14 years); 9 patients were male (56.2%), and 7 were female. The mean age was of 55.3 (range: 32–78) years. In 75% of the cases, the DS was affected, and 11 patients (68.8%) practiced sports. In two cases, there was history of trauma (in these cases, acromioplasty had not been performed), and in four cases, there was a diagnosis of SLAP injury, for which tenotomy and tenodesis were performed in wo cases, and in two cases, debridement.

**Fig. 1:**
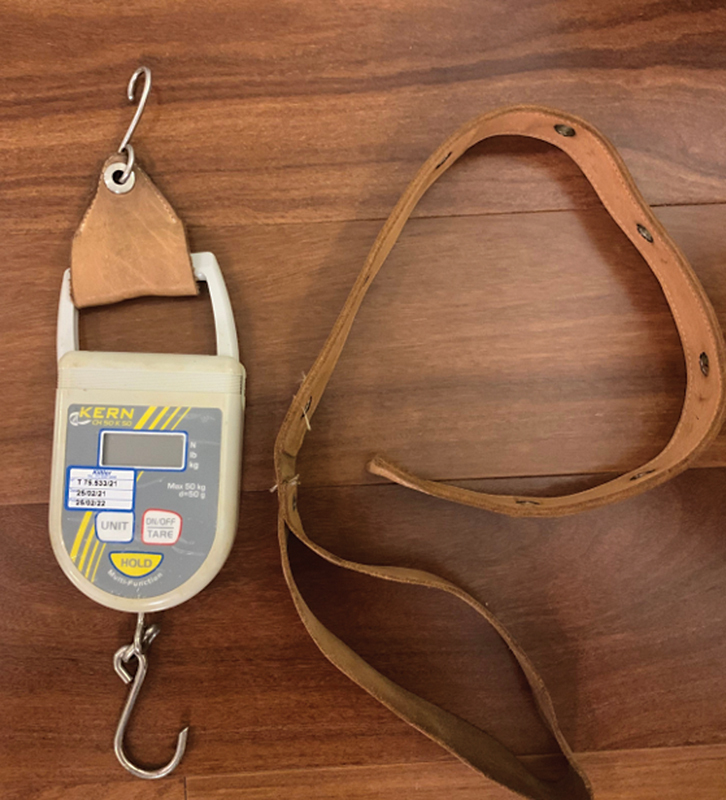
Front photo of the properly-calibrated Kern CH50 K 50 digital dynamometer, which measures isometric shoulder force.

**Fig. 2:**
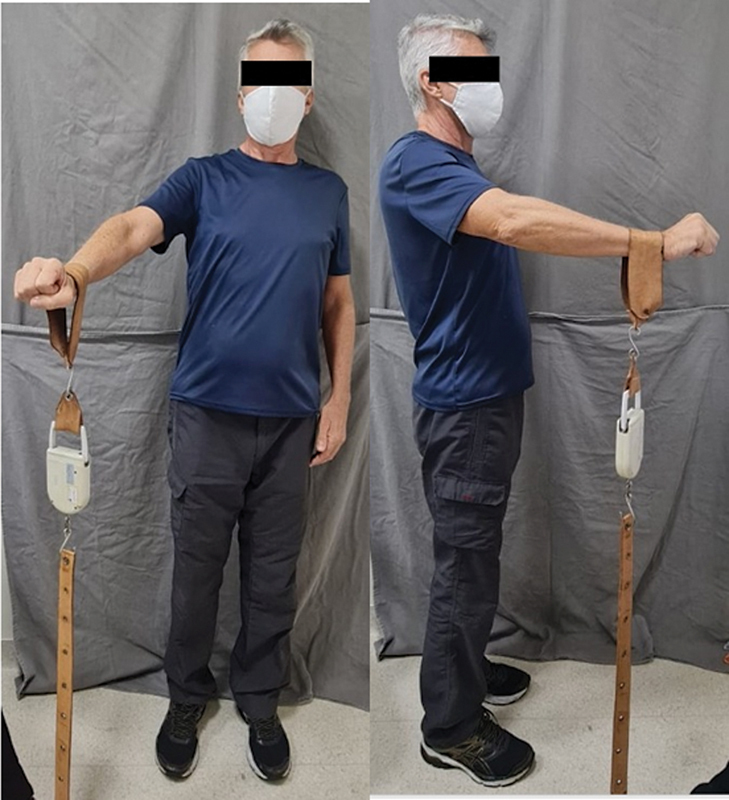
Patient performing an isometric strength test of the right shoulder using the properly-calibrated digital dynamometer KERN CH50 K 50.

After collecting the data from groups III and IV, we calculated the scores on the modified University of California, Los Angeles (UCLA) Shoulder Rating Scale, 26 the Constant-Murley score, 27 and the strength of the operated shoulder of all patients. To measure the isometric contraction force, the KERN CH50 K 50 digital dynamometer (Kern & Sohn GmbH, Balingen, Germany) was used ( Fig.1 ), calibrated according to the primary standards of the Brazilian Calibration Network (Rede Brasileira de Calibração, RBC, in Portuguese) of the Brazilian National Institute of Metrology, Quality and Technology (Instituto Nacional de Metrologia, Qualidade e Tecnologia, INMETRO, in Portuguese), as recommended by the Constant-Murley score ( Fig. 2 ).

The Anderson-Darling, Ryan-Joiner, and Kolmogorov-Smirnov tests were used to determine the normality of the distributions. When the distribution was normal, the Student *t* -test was used to calculate the confidence intervals for the mean, and the Fisher F-test was used to compare the variances. Otherwise, Wilcoxon confidence intervals were calculated, and the Mann-Whitney test was used to analyze the means. The means of the variables strength (kg), current UCLA score, and Constant-Murley score regarding sex, age, and dominance were then analyzed and compared between groups III and IV. To test, in the interval of both evaluations, if there was a change in the mean UCLA score between groups I and III and between groups II and IV, the Wilcoxon test was used.

The analyses were performed using the Minitab Statistical Software, version 21 (Minitab, LLC, State College, PA, USA), and all hypotheses with descriptive levels ( *p* -value) lower than 0.05 were rejected.

The present work was submitted to the institutional Research Ethics Committee and approved according to CAAE: 79428617.2.0000.5479.

## Results

Regarding group III, the mean UCLA score was of 33.8, with 12 excellent results (66.7%), 6 good results (33.3%), and no unsatisfactory results. There was no statistical difference between the mean current UCLA score according to sex ( *p* = 0.277), affection or not of the DS, ( *p* = 0.755), or age ( *p* = 0.755). The mean Constant-Murley score was of 91.9, with 13 excellent results (72.2%), 4 good results (22.2%), and 1 satisfactory result (score of 72; case 11) that lost points in the mobility and strength questions and presented 31 points on the current UCLA scale, losing points in the function analysis ( Table 1 ). There was no statistical difference between the mean Constant-Murley score according to sex ( *p* = 0.962), affection or not of the DS, ( *p* = 0.254), or age ( *p* = 0.404). Concerning the mean strength, there was a statistical difference in cases of DS affection ( *p* = 0.023); therefore, it is possible to say that the DS presented a higher mean strength (11 kg) compared to the non-dominant side (NDS; 7.8 kg).

**Table 1:** Clinical data after the second evaluation of patients undergoing transtendon repair (TTR); group III

PN	Case	Sex	Age (years)	Dom	Δtt (years)	UCLA	Strength (kg)	Rounded strength (P)	Constant-Murley Score
1	1	F	53		6	35	11	24	97
2	2	M	74		6	35	9	20	91
3	3	F	52	+	6	33	12	25	97
4	4	F	43	+	6	35	10	22	93
5	5	M	54	+	6	35	12	25	99
6	7	M	53	+	6	35	10	22	91
7	8	M	43	+	12	31	10	22	90
8	9	M	38	+	6	35	10	22	93
9	10	M	47	+	12	34	12	25	90
10	11	F	59		12	31	4	9	72
11	12	M	44	+	15	35	12	25	96
12	13	F	58	+	6	33	11	24	99
13	14	M	56	+	13	32	9	20	90
14	15	M	62		8	34	6	13	84
15	16	M	62	+	12	34	11	24	93
16	17	F	56		8	33	9	20	91
17	18	M	47	+	8	34	12	25	95
18	19	M	38	+	10	35	12	25	94

**Abbreviations:** Δtt, postoperative follow-up time; Dom, dominance; F, female; kg, kilograms; P, pounds; M, male; PN, patient number; UCLA, modified University of California, Los Angeles Shoulder Rating Scale.

Regarding group IV, the mean UCLA score was of 32.9, with 6 excellent results (37.5%), 9 good results (56.2%), and 1 unsatisfactory result (6.3%). In the second evaluation, a case that had presented an excellent result in the first evaluation 21 (case 8), with a score of 35, had its score decreased to 27 mainly due to pain and decreased function and strength after falling to the ground and trauma 8 months earlier. Due to the coronavirus disease 2019 (COVID-19) pandemic, the patient did not want to seek medical assistance ( Table 2 ). There was no statistical difference between the mean current UCLA score according to sex ( *p* = 0.298), affection or not of the DS ( *p* = 0.781), or age ( *p* = 1.000). The mean Constant-Murley score was of 86.8, with 6 excellent results (37.5%), 8 good results (50%), 1 satisfactory result (6.25%), and 1 regular result (6.25%). The patient with the satisfactory result on the Constant-Murley score (case 9) lost points in the strength question (9 points), although they remained with a satisfactory UCLA score, of 33 points. The case with a regular score (case 8) was the same one that presented a low UCLA score due to the fall suffered 8 months earlier. There was a statistical difference between the mean Constant-Murley score according to sex, strength, and age, and, proportionally, men ( *p* = 0.012) under 60 years of age ( *p* = 0.043) obtained, in a statistically significant way ( *p* = 0.003), a higher score (mean of 91.6) mainly due to greater strength (mean strength of 10.4 kg). Concerning the mean strength, there was no statistical difference in terms of age ( *p* = 0.059), despite the *p* -value being very close to the significance level, showing a trend among elderly patients (over 60 years old) to present lower mean strength (5.5 kg) compared to younger patients (9.3 kg). Regarding the mean force and whether or not the DS was affected, there was no statistical difference ( *p* = 0.129), with the mean force of 8.25 kg on the DS against 6.75 kg on the NDS.

**Table 2:** Clinical data after the second evaluation of patients undergoing repair after completing the lesion (TCR); group IV

PN	Case	Sex	Age (years)	Dom	Δtt (years)	UCLA	Strength (kg)	Rounded strength (P)	Constant-Murley Score	COMP
1	1	M	62	+	6	35	12	25	98	
2	4	F	71	+	6	33	4	9	82	
3	5	M	78		6	34	7	15	82	
4	6	F	60		8	33	4	9	82	
5	7	M	42	+	11	34	10	22	91	
6	8	F	76	+	8	27	2	4	65	T
7	9	F	63	+	6	33	4	9	78	
8	11	M	42	+	6	33	12	25	94	
9	12	F	32	+	14	33	10	22	93	
10	14	M	46	+	6	33	10	22	88	
11	15	F	54		11	32	4	9	82	
12	16	M	42	+	9	32	9	20	87	
13	17	M	53	+	6	31	10	22	88	
14	18	M	54	+	6	34	10	22	95	
15	19	M	59		6	35	12	25	98	
16	20	F	51	+	6	35	6	13	86	

**Abbreviations:** Δtt, postoperative follow-up time; COMP, complication; Dom, dominance; F, female; kg, kilograms; P, pounds; M, male; PN, patient number; T, Trauma; UCLA, modified University of California, Los Angeles Shoulder Rating Scale.

When comparing groups III and IV, we did not notice statistical differences in relation to sex ( *p* = 0.533), age ( *p* = 0.488), and dominance ( *p* = 0.693), showing that both groups were statistically similar. When the mean UCLA score was compared between them, there was no statistical difference ( *p* = 0.113), with a mean total final result of 33.8 for group III (range: 31–35) and of 32.9 for group IV (range: 27–35). When the mean Constant-Murley score was compared between them, there was no statistical difference ( *p* = 0.075), with a mean final result of 91.9 for group III (range: 72–99) and of 86.8 for group IV (range: 65–98) ( Table 3 ).

**Table 3:** Comparison of the mean UCLA and Constant-Murley scores of groups III and IV

	Group III	Group IV	*p* -value
UCLA	33.8	32.9	0.113
Constant-Murley	91.9	86.8	0.075

**Abbreviation:** UCLA, modified University of California, Los Angeles Shoulder Rating Scale.

When comparing the mean DS strength between groups III and IV, there was a statistical difference: group III presented a higher mean force (11 kg) than group IV (8.25 kg), with *p* = 0.042.

When evaluating patients subjected to the TTR technique, groups I 21 and III, the mean previous and current UCLA scores were, and no statistical difference was observed ( *p* = 0.374) ( Table 4 ).

**Table 4:** Comparison of the UCLA score on the evaluations of patients operated on through the TTR technique

PN	Case	UCLA 1 21	UCLA 2 (second evaluation)	*p* -value
1	1	35	35	
2	2	35	35	
3	3	35	33	
4	4	35	35	
5	5	35	35	
6	7	35	35	
7	8	32	31	
8	9	35	35	
9	10	32	34	
10	11	31	31	
11	12	34	35	
12	13	32	33	
13	14	31	32	
14	15	23	34	
15	16	34	34	
16	17	33	33	
17	18	33	34	
18	19	33	35	
Average		32.9	33.8	0.374

**Abbreviations:** PN, patient number; TTR, transtendon repair; UCLA, modified University of California, Los Angeles Shoulder Rating Scale .

When evaluating patients subjected to the TCR technique, groups II 21 and IV, the mean previous and current UCLA scores were compared, and no statistical difference was observed ( *p* = 0.859) ( Table 5 ).

**Table 5:** Comparison of the UCLA score on the evaluations of patients operated on with the TCR technique

PN	Case	UCLA 21	UCLA (second evaluation)	*p* -value
1	1	32	35	
2	4	33	33	
3	5	34	34	
4	6	34	33	
5	7	32	34	
6	8	35	27	
7	9	32	33	
8	11	33	33	
9	12	34	33	
10	14	32	33	
11	15	33	32	
12	16	32	32	
13	17	33	31	
14	18	32	34	
15	19	33	35	
16	20	35	35	
Average		33.1	32.9	0.859

**Abbreviations:** PN, patient number; TCR, tear completion repair; UCLA, modified University of California, Los Angeles Shoulder Rating Scale.

## Discussion

Regarding the TCR technique, Shin (2012) mentions that the disinsertion of the entire lateral margin of the rotator cuff by transforming the partial injury into a complete one can lead to the risk of non-anatomical repair, altering the biomechanics and thus causing an early degeneration of the tendon, which can increase the rates of rerupture. 16 Ono et al. 20 show that the postrepair rupture index varies on average from 1.8 to 6.1% according to the technique used, with the techniques being TTR and TCR respectively, but the final results were good/excellent regardless of the techniques and after 40.5 months of follow-up. In the present study, we may have a case of posttraumatic rupture (case 8, group IV), an event unrelated to the procedure performed, but which could not be confirmed because the patient did not want to seek medical assistance. Apart from this case, we have no clinical suspicion of any case that could have reruptured once, since, according to the UCLA scale, as all cases fall between good and excellent.

The literature shows the relationship involving the results obtained according to the scores studied and the time of postoperative evolution. Few studies have extensive postoperative follow-up, longer than 2 years. 1 23 24 25 Stuart et al. 1 evaluated patients with PTRCT in 2 postoperative moments (1 and 12 years) and noticed that there was no change between evaluations after the statistically significant improvement in the first evaluation. Vap et al. 23 showed that, after 5 years after PTRCT, the functional and patient satisfaction results were excellent, corroborating the idea that these results remain high in the medium term. Rossi et al. 24 observed excellent results in more than 80% of their patients with PTRCT after a minimum of 8 years of postoperative follow-up. Similarly, Dey Hazra et al. 25 performed 3 postoperative reevaluations of their patients with PTRCT (2, 5, and 10 years) and noticed that, after the improvement in the first evaluation, the result remained without statistical difference. In the present work, the same thing occurred as in the aforementioned studies. Sella et al. 21 observed mostly good and excellent results 2 years postoperatively, and in the second evaluation, at a minimum of 6 years of postoperative follow-up, the evaluation parameters were not statistically altered.

Clinically, when performing this current evaluation in our patients, we noticed a significant increase ( *p* = 0.042) in the mean strength of patients in group III (DS: 11 kg,; NDS: 7.8 kg) in relation to group IV (DS: 8.25 kg; NDS: 6.75 kg), although no statistical difference was observed between the Constant-Murley scores. 27 In our opinion, it is highly likely that these higher values are due to the fact that part of the tendon remains native to its original insertion in the TTR technique. None of the articles that perform the clinical comparison between the methods report that there was a statistical difference between the final forces found, since they do not discuss the force variable, although all use the Constant-Murley score in their evaluations. 8 11 16 17 18 20 22 It is understood that the strength variable improves regardless of the surgical method chosen because the vast majority of studies affirm that there is an improvement in the score. This is confirmed when we analyze separately those articles that use only one surgical technique in their study and that evaluate the final strength of their patients in some way. Castagna et al. 28 clinically evaluated only patients subjected to the TTR technique. They describe that they used a muscle test system named Lafayette, but the results do not specify values, and they only report a significant increase in the Constant-Murley score, and that all parameters of this score improved. For example, Fama et al. 19 obtained a significant increase in patients' final strength when using the TCR technique. They creported preoperative and postoperative mean strength values of 4.4 kg and 9.9 kg, but did not specify whether the shoulder in question was on the DS or the NDS. 19

We understand that the follow-up of patients for longer periods always helps us to better understand the long-term evolution. Compared to our previous work, 21 we almost doubled the follow-up time, but mainly increased the minimum follow-up time to 6 years. Regarding the few studies with long-term follow-up, our minimum follow-up was higher than the one adopted by Vap et al. 23 The present is the first study published so far, in English, in which there is a clinical comparison of the techniques used specifically discussing the strength variable, which was even statistically significant in favor of patients undergoing the TTR technique. We consider this a strong point and an important factor for dissemination among experts.

As a definition of conduct, our group chooses to repair PTRCT by the TTR technique in young patients whose injury is of traumatic origin and in those who perform sports that require upper limb strength, since this is the only variable that obtains statistically significant improvement. In elderly patients whose injury is of degenerative origin and who do not require substantial strength gain for sports practice, the TCR is chosen due to technical ease and reduced surgical time. This technique will certainly bring significant improvement in shoulder pain and function.

## Conclusion

We conclude that the arthroscopic treatment of PTRCT, both by the TTR and the TCR techniques, resulted in satisfactory mean UCLA scores, with no statistical difference. We also obtained satisfactory mean Constant-Murley scores for both techniques, with no statistical difference between them. Considering the same technique used, neither was there statistical difference between the first and second evaluations.

After 6 years, patients treated through the TTR technique achieved statistically higher mean DS strength compared to their NDS and to the patients treated through the TCR technique.
