# Supplementary material for: Comparative Clinical Evaluation of Two Techniques in the Arthroscopic Treatment of Partial Articular Rotator Cuff Tears after Six Years of Follow-up
Source: Rev Bras Ortop (Sao Paulo). 2024 Dec 7;59(5):e771–8. doi: 10.1055/s-0044-1790211 (PMC11624924; doi:10.1055/s-0044-1790211)
Supplement: Related texts

# Research article: Avaliação clínica comparativa de duas técnicas no tratamento artroscópico das lesões parciais articulares do manguito rotador após seis anos de seguimento

**Keywords:** artroscopia, manguito rotador, tratamento

## Abstract

**Objetivo** Avaliar clinicamente a médio prazo os resultados do tratamento artroscópico das lesões parciais da porção articular do manguito rotador (LPPAMR) pelas técnicas de reparo transtendão (RTT) e reparo após completada a lesão (RPCL) utilizando-se a Escala Modificada de Avaliação do Ombro da University of California, Los Angeles (UCLA), a escala de Constant-Murley e análise de força.

**Métodos** Estudo retrospectivo de reavaliação, após seguimento mínimo de 6 anos, de casos de LPPAMR operados artroscopicamente, envolvendo um total de 34 pacientes, sendo 18 submetidos ao RTT e 16 ao RPCL. Foram comparadas as pontuações atuais nas escala da UCLA e de Constant-Murley, a força média entre as técnicas, e as pontuações na UCLA atualmente e com 2 anos de pós-operatório do mesmo grupo, conforme publicado em estudo anterior, a fim de avaliarmos se houve ou não modificação dos resultados ao longo do tempo.

**Resultados** Não houve diferença estatística entre as pontuações nas escalas. As pontuações na escala da UCLA atual foram de 33,8 para o RTT e de 32,9 para o RPCL ( *p* = 0,113), e na escala de Constant-Murley, foram de 91,9 e 86,8, respectivamente ( *p* = 0,075). Para o RTT, a pontuação anterior na escala da UCLA (com 2 anos de pós-operatório) foi 32,4, e a atual, de 33,8 ( *p* = 0,374); para o RPCL, foi de 32,4 com 2 anos de pós-operatório, e de 32,9 atualmente ( *p* = 0,859). No RTT, a força média foi estatisticamente maior do lado dominante (11 kg) do que no não dominante (7,80 kg) ( *p* = 0,023) e aqueles do RPCL (8,25 kg) ( *p* = 0,042).

**Conclusão** Não houve diferença estatística em médio prazo na comparação das pontuações nas escalas da UCLA e de Constant-Murley em relação à técnica utilizada (RTT ou RPCL), nem alteração nas pontuações na escala da UCLA entre 2 e 6 anos de pós-operatório. Nos pacientes submetidos à RTT, a força média foi estatisticamente maior do lado dominante do que no não dominante, e maior do que nos pacientes submetidos à RPCL.

## Introdução

A lesão parcial do manguito rotador é uma importante causa de disfunção e dor no ombro. 1 Sua incidência ainda não é consenso na literatura apesar de existirem estudos 2 3 4 que evidenciam valores de 14,5 4 a 32%. 3 Fukuda ainda descreve que aproximadamente 1/3 (27%) dessas lesões acometem a porção articular. 4

Ellman foi o primeiro a propor, baseado em achados artroscópicos, um sistema de classificação da lesão parcial em relação a sua localização (A: articular; B: bursal; C: intratendínea) e sua extensão (grau 1: < 3 mm; grau 2: 3–6 mm; grau 3: > 6 mm). 5

Quanto à fisiopatologia das lesões parciais da porção articular do manguito rotador (LPPAMR), acredita-se que podem ocorrer tanto com mecanismos intrínsecos (área de hipovascularização e alterações metabólicas relativas à idade), quanto extrínsecos (impacto interno póstero superior, eventos traumáticos agudos e microtraumas de repetição), ou até mesmo, uma combinação deles. 6 7

O tratamento inicial da LPPAMR é realizado com medidas conservadoras, como fisioterapia e mudança dos hábitos para evitar a progressão da lesão, 8 e o tratamento cirúrgico está indicado na falha deste ou nos casos em que a lesão acometa mais de 50% da espessura do tendão, devido um risco de até 40% na sua progressão. 1 9

Diferentes técnicas cirúrgicas artroscópicas têm sido descritas para abordagem desse tipo de lesão, como um simples desbridamento artroscópico do tendão, 2 6 10 11 12 ou reparo deste. Como reparo, utiliza-se técnica de reparo transtendão (RTT), descrita inicialmente por Snyder, 13 ou ainda o reparo após completar a lesão (RPCL). 8 11 14 15 16 17 18 19 Existem trabalhos com avaliações a curto prazo comparando qual técnica cirúrgica seria a melhor clinicamente para o tratamento das LPPAMR 8 16 17 18 20 21 22 e todos chegaram à conclusão de que não há diferença estatística entre eles. Alguns poucos trabalhos 1 23 24 25 realizam esta comparação a longo e médio prazo. O objetivo deste trabalho é reavaliar os pacientes operados por ambas as técnicas supracitadas, cujos resultados foram publicados anteriormente, 21 agora com seguimento no médio prazo e com análise da força atual, a fim de avaliar se houve mudança nos resultados.

## Materiais e Métodos

De outubro de 1999 a dezembro de 2016, 39 pacientes foram submetidos a tratamento cirúrgico artroscópico com diagnóstico de LPPAMR pelo Grupo de Ombro da nossa instituição. Todos os casos operados foram classificados como Ellman 3 (lesão parcial superior a 50% da espessura tendinosa). Em nenhum dos casos foi realizado apenas o debridamento cirúrgico, e o reparo da lesão foi realizado em todos os casos. Foram utilizadas duas técnicas de reparo artroscópico, a transtendão (RTT) em 19 pacientes (grupo I), e o reparo após completada a lesão (RPCL) em 20 pacientes (grupo II). A escolha da técnica cirúrgica ficou a cargo do cirurgião, e a avaliação foi retrospectiva. Foram avaliados e comparados clinicamente em estudo prévio 21 após seguimento mínimo de 2 anos.

Para este estudo foi realizado contato com 18 pacientes (94,7% do grupo inicial) operados pela técnica RTT (denominado grupo III) e 16 pacientes (80% do grupo inicial) pela técnica RPCL (denominado grupo IV).

O grupo III teve média de seguimento de 105,6 meses (8,8 anos; variação: 6–15 anos), e 12 pacientes eram do sexo masculino (66,7%) e 6 do sexo feminino. A média de idade foi 52,2 (variação: 38–74) anos. O lado dominante (LD) foi acometido em 72,2% dos casos, e 15 pacientes (83,3%) praticavam esporte. Em 9 casos havia histórico de trauma (nestes não fora realizada acromioplastia) e em 6 casos havia diagnóstico de lesões do lábrum superior, de anterior para posterior ( *superior labral anterior to posterior* , SLAP, na sigla em inglês): foram realizadas tenotomia e tenodese em 5 casos e, em 1 caso, debridamento.

O grupo IV teve média de seguimento de 91,2 meses (7,6 anos; variação: 6–14 anos), e 9 pacientes eram do sexo masculino (56,2%) e 7 do sexo feminino. A média de idade foi 55,3 (variação: 32–78) anos. O LD foi acometido em 75% dos casos e 11 pacientes (68,8%) praticavam esporte. Em dois casos havia histórico de trauma (nestes não fora realizada acromioplastia) e em quatro casos havia diagnóstico de SLAP (foram realizadas tenotomia e tenodese em dois casos e em dois casos, debridamento).

Após a coleta dos dados dos grupos III e IV, foram calculadas as pontuações na Escala Modificada de Avaliação do Ombro da University of California, Los Angeles (UCLA) 26 e na escala de Constant-Murley, 27 e a força do ombro operado de todos os pacientes. Para a mensuração da força de contração isométrica, foi utilizado o dinamômetro digital KERN CH50 K 50 (Kern & Sohn GmbH, Balingen, Alemanha) ( Fig. 1 ), calibrado de acordo com os padrões primários da Rede Brasileira de Calibração (RBC) do Instituto Nacional de Metrologia, Qualidade e Tecnologia (INMETRO), conforme preconiza a escala de Constant-Murley ( Fig. 2 ).

**Fig. 1:** Foto de frente do dinamômetro digital KERN CH50 K 50, devidamente calibrado, utilizado para mensuração de força isométrica do ombro.

**Fig. 2:** Paciente realizando teste de força isométrica do ombro direito, utilizando o dinamômetro digital KERN CH50 K 50, devidamente calibrado.

Foram utilizados os testes de Anderson-Darling, Ryan-Joiner e Kolmogorov-Smirnov para testar a normalidade das distribuições. Nos casos de distribuição normal, utilizou-se o teste *t* de Student no cálculo dos intervalos de confiança para média ou o teste F de Fisher para comparação das variâncias. Caso contrário, foram calculados os intervalos de confiança de Wilcoxon e para análise das médias, o teste de Mann-Whitney. Foram então analisadas as médias das variáveis força (kg), a pontuação atual de UCLA e a pontuação de Constant-Murley em função do sexo, idade e dominância, e foram comparados os resultados dos grupos III e IV. O teste de Wilcoxon foi utilizado para avaliar, no intervalo de ambas as avaliações, se houve alteração na média da pontuação na escala da UCLA entre os grupos I e III e os grupos II e IV.

As análises foram realizadas utilizando o programa estatístico Minitab Statistical Software, versão 21 (Minitab, LLC, State College, PA, EUA), e todas as hipóteses com níveis descritivos (valor de *p* ) inferiores a 0,05 foram rejeitadas.

Este trabalho foi submetido ao Comitê de Ética em Pesquisa de nossa instituição e aprovado conforme CAAE: 79428617.2.0000.5479.

## Resultados

Com relação ao grupo III, a pontuação média da UCLA foi 33,8, sendo 12 resultados excelentes (66,7%), 6 resultados bons (33,3%) e nenhum resultado insatisfatório. Não houve diferença estatística entre a média da UCLA atual em função do sexo ( *p* = 0,277), do acometimento ou não do LD ( *p* = 0,755), ou da idade ( *p* = 0,755). Em relação à pontuação na escala de Constant-Murley, a média foi 91,9, sendo 13 resultados excelentes (72,2%), 4 resultados bons (22,2%) e 1 resultado satisfatório (pontuação 72; caso 11), que perdeu pontos nos quesitos mobilidade e força e que apresentou 31 pontos na escala da UCLA atual, perdendo pontos na análise da função ( Tabela 1 ). Não houve diferença estatística entre a pontuação média de Constant-Murley em função do sexo ( *p* = 0,962), do acometimento ou não do LD ( *p* = 0,254), ou da idade ( *p* = 0,404). Em relação à força média, houve diferença estatística em relação ao acometimento do LD ( *p* = 0,023); portanto, pode-se dizer que o LD apresentou força média maior (11 kg) em comparação com o lado não dominante (LND; 7,8 kg).

**Tabela 1:** Dados clínicos após a segunda avaliação dos pacientes submetidos à técnica de reparo transtendão (RTT), grupo III

NP	Caso	Sexo	Idade (anos)	Dom	Δtt (anos)	UCLA	Força (kg)	Força arredondada (L)	Escala de Constant-Murley
1	1	F	53		6	35	11	24	97
2	2	M	74		6	35	9	20	91
3	3	F	52	+	6	33	12	25	97
4	4	F	43	+	6	35	10	22	93
5	5	M	54	+	6	35	12	25	99
6	7	M	53	+	6	35	10	22	91
7	8	M	43	+	12	31	10	22	90
8	9	M	38	+	6	35	10	22	93
9	10	M	47	+	12	34	12	25	90
10	11	F	59		12	31	4	9	72
11	12	M	44	+	15	35	12	25	96
12	13	F	58	+	6	33	11	24	99
13	14	M	56	+	13	32	9	20	90
14	15	M	62		8	34	6	13	84
15	16	M	62	+	12	34	11	24	93
16	17	F	56		8	33	9	20	91
17	18	M	47	+	8	34	12	25	95
18	19	M	38	+	10	35	12	25	94

**Abreviaturas:** Δtt, tempo de seguimento pós-operatório; Dom, dominância; F, feminino; kg, quilogramas; L, libras; M, masculino; NP, número do paciente; UCLA, Escala Modificada de Avaliação do Ombro da University of California, Los Angeles.

Com relação ao grupo IV, a média na UCLA foi de 32,9, sendo 6 resultados excelentes (37,5%), 9 resultados bons (56,2%) e 1 resultado insatisfatório (6,3%). No momento da segunda avaliação, um caso que havia resultado excelente na primeira avaliação 21 (caso 8), com pontuação de 35 na UCLA, teve sua pontuação diminuída para 27, principalmente devido a dor e diminuição de função e força após queda ao solo e trauma havia 8 meses. Devido à pandemia de doença do coronavírus 2019 ( *coronavirus disease 2019* , COVID 19, em inglês), o paciente não quis procurar assistência médica ( Tabela 2 ). Não houve diferença estatística com relação á média atual na UCLA em função do sexo ( *p* = 0,298), do acometimento ou não do LD ( *p* = 0,781), ou da idade ( *p* = 1,000). Em relação à escala de Constant-Murley, a média foi 86,8, sendo 6 resultados excelentes (37,5%), 8 resultados bons (50%), 1 resultado satisfatório (6,25%) e 1 resultado regular (6,25%). O resultado satisfatório na escala de Constant-Murley (caso 9) perdeu pontos no quesito força (9 pontos), apesar de manter-se com pontuação satisfatória na UCLA (33 pontos). O caso pontuado como regular (caso 8) foi o mesmo que apresentou pontuação baixa na UCLA devido à queda sofrida havia 8 meses. Houve diferença estatística entre a média na escala de Constant-Murley em função do sexo, da força e da idade, sendo que, proporcionalmente, os homens ( *p* = 0,012) com idade inferior a 60 anos ( *p* = 0,043) obtiveram, de forma estatisticamente significativa ( *p* = 0,003), uma pontuação maior (média de 91,6), principalmente devido à maior força (média: 10,4 kg). Em relação à força média, não houve diferença estatística em relação à idade ( *p* = 0,059), apesar de o valor de *p* ter ficado muito próximo do nível de significância, o que mostra uma tendência dos pacientes idosos (acima de 60 anos) a ter menor força média (5,5 kg) em relação aos mais jovens (9,3 kg). Em relação à força média e ao lado ser ou não o dominante, não houve diferença estatística ( *p* = 0,129), sendo a média da força de 8,25 kg no LD contra 6,75 kg no LND.

**Tabela 2:** Dados clínicos após a segunda avaliação dos pacientes submetidos à técnica de reparo após completada a lesão (RPCL), grupo IV

NP	Caso	Sexo	Idade (anos)	Dom	Δtt (anos)	UCLA	Força (kg)	Força arredondada (L)	Escala de Constant-Murley	Comp.
1	1	M	62	+	6	35	12	25	98	
2	4	F	71	+	6	33	4	9	82	
3	5	M	78		6	34	7	15	82	
4	6	F	60		8	33	4	9	82	
5	7	M	42	+	11	34	10	22	91	
6	8	F	76	+	8	27	2	4	65	T
7	9	F	63	+	6	33	4	9	78	
8	11	M	42	+	6	33	12	25	94	
9	12	F	32	+	14	33	10	22	93	
10	14	M	46	+	6	33	10	22	88	
11	15	F	54		11	32	4	9	82	
12	16	M	42	+	9	32	9	20	87	
13	17	M	53	+	6	31	10	22	88	
14	18	M	54	+	6	34	10	22	95	
15	19	M	59		6	35	12	25	98	
16	20	F	51	+	6	35	6	13	86	

**Abreviaturas:** Δtt, tempo de seguimento pós-operatório; COMP, complicação; Dom, dominância; F, feminino; kg, quilogramas; L, libras; M, masculino; NP, número do paciente; T, trauma; UCLA, Escala Modificada de Avaliação do Ombro da University of California, Los Angeles.

Ao compararmos os grupos III e IV, não notamos diferença estatística em relação a sexo ( *p* = 0,533), idade ( *p* = 0,488), e dominância ( *p* = 0,693), sendo ambos os grupos estatisticamente semelhantes. Comparando a pontuação média na UCLA entre eles, não houve diferença estatística ( *p* = 0,113), com resultado final total médio de 33,8 para o grupo III (31–35) e de 32,9 para o grupo IV (27–35). Comparando a pontuação média de Constant-Murley entre os grupos, não houve diferença estatística ( *p* = 0,075), com resultado final médio de 91,9 para o grupo III (variação: 72–99) e de 86,8 para o grupo IV (variação: 65–98) ( Tabela 3 ).

**Tabela 3:** Comparação das pontuações médias de UCLA e de Constant-Murley dos grupos III e IV

	Grupo III	Grupo IV	Valor de *p*
UCLA	33,8	32,9	0,113
Escala de Constant-Murley	91,9	86,8	0,075

**Abreviatura:** UCLA, Escala Modificada de Avaliação do Ombro da University of California, Los Angeles.

Ao compararmos a força média do LD entre os grupos III e IV, houve diferença estatística, sendo que o grupo III apresentou força média maior (11 kg) do que o grupo IV (8,25 kg), com *p* = 0,042.

Ao avaliarmos os pacientes submetidos à técnica de RTT, os grupos I 21 e III, comparou-se a média anterior na UCLA com a média atual, e não houve diferença estatística ( *p* = 0,374) ( Tabela 4 ).

**Tabela 4:** Comparação da pontuação de UCLA entre as avaliações dos pacientes operados pela técnica de RTT

NP	Caso	UCLA 1 21	UCLA 2 (2ª avaliação)	Valor de *p*
1	1	35	35	
2	2	35	35	
3	3	35	33	
4	4	35	35	
5	5	35	35	
6	7	35	35	
7	8	32	31	
8	9	35	35	
9	10	32	34	
10	11	31	31	
11	12	34	35	
12	13	32	33	
13	14	31	32	
14	15	23	34	
15	16	34	34	
16	17	33	33	
17	18	33	34	
18	19	33	35	
Média		32,9	33,8	0,374

**Abreviaturas:** NP, número do paciente; RTT, reparo transtendão; UCLA, Escala Modificada de Avaliação do Ombro da University of California, Los Angeles.

Ao avaliarmos os pacientes submetidos à técnica de RPCL, os grupos II 21 e IV, comparou-se a média anterior na UCLA com a média atual, e não houve diferença estatística ( *p* = 0,859) ( Tabela 5 ).

**Tabela 5:** Comparação da pontuação de UCLA entre as avaliações dos pacientes operados pela técnica de RPCL

NP	Caso	UCLA 21	UCLA (2 ^a^ avaliação)	Valor de *p*
1	1	32	35	
2	4	33	33	
3	5	34	34	
4	6	34	33	
5	7	32	34	
6	8	35	27	
7	9	32	33	
8	11	33	33	
9	12	34	33	
10	14	32	33	
11	15	33	32	
12	16	32	32	
13	17	33	31	
14	18	32	34	
15	19	33	35	
16	20	35	35	
Média		33,1	32,9	0,859

**Abreviaturas:** NP, número do paciente; RPCL, reparo após completada a lesão; UCLA, Escala Modificada de Avaliação do Ombro da University of California, Los Angeles.

## Discussão

Sobre a técnica RPCL, Shin (2012) afirma que a desinserção de toda a margem lateral do manguito rotador, ao transformar a lesão parcial em completa, pode levar ao risco do reparo não anatômico, o que altera a biomecânica e, portanto, provoca uma degeneração precoce do tendão, o que pode aumentar os índices de rerrotura. 16 Ono et al. 20 evidenciam que o índice de rotura pós-reparo varia em média de 1,8 a 6,1% de acordo com a técnica utilizada, sendo as técnicas RTT e RPCL, respectivamente; porém, os resultados finais foram bons/excelentes independentemente da técnica e após 40,5 meses de seguimento. Neste estudo, temos um possível caso de rerrotura pós-traumática (caso 8, grupo IV), evento este não relacionado com o procedimento realizado, mas que não pôde ser confirmado porque a paciente não quis procurar assistência médica. Além desse caso, não desconfiamos clinicamente de nenhum caso de rerrotura, uma vez que, de acordo com a escala da UCLA, todos os casos se enquadram entre bons e excelentes.

A literatura nos mostra a relação entre os resultados obtidos de acordo com as escalas estudadas e o tempo de evolução pós-operatória. Poucos trabalhos têm seguimento pós-operatório extenso, mais longo do que 2 anos. 1 23 24 25 Stuart et al. 1 avaliaram os pacientes com LPPAMR em 2 momentos pós-operatórios (1 e 12 anos), e perceberam que, após a melhora estatisticamente significativa na primeira avaliação, não houve alteração entre as avaliações. Vap et al. 23 evidenciaram que, aos 5 anos de pós-operatório da LPPAMR, os resultados funcionais e de satisfação dos pacientes foram excelentes, o que corrobora a ideia de que estes resultados se mantêm elevados no médio prazo. Rossi et al. 24 identificaram resultados excelentes em mais de 80% de seus pacientes com LPPAMR após um seguimento pós-operatório mínimo de 8 anos. Da mesma forma, Dey Hazra et al. 25 realizaram 3 reavaliações pós-operatórias dos seus pacientes com LPPAMR (2, 5 e 10 anos), e também perceberam que, após a melhora registrada na primeira avaliação, o resultado se manteve, sem diferença estatística. Neste trabalho, ocorreu a mesma coisa do que nesses estudo citados anteriormente. Sella et al. 21 observaram maioria de resultados bons e excelentes após 2 anos de pós-operatório e, na segunda avaliação, após um seguimento pós-operatório mínimo de 6 anos, não houve modificação estatística dos parâmetros de avaliação.

Clinicamente, ao realizarmos esta avaliação atual em nossos pacientes, percebemos o aumento significativo ( *p* = 0,042) da força média dos pacientes do grupo III (LD: 11 kg; LND: 7,8 kg) em relação ao grupo IV (LD: 8,25 kg; LND: 6,75 kg), embora não tenha havido diferença estatística entre as pontuações na escala de Constant-Murley. 27 Na nossa opinião, é bastante provável que esses valores maiores se devam ao fato de que se mantém parte do tendão nativo à sua inserção original na técnica de RTT. Nenhum dos artigos que fez a comparação clínica entre os métodos relatou que houve diferença estatística entre as forças finais encontradas, pois não consideraram a variável força, apesar de todos terem utilizado a escala de Constant-Murley em suas avaliações. 8 11 16 17 18 20 22 Subentende-se que a variável força melhore independentemente do método cirúrgico escolhido, pois a maioria dos estudos afirmam que há melhora na pontuação na escala. Isso se confirma quando analisamos separadamente aqueles estudos que utilizaram apenas uma técnica cirúrgica e que avaliaram de alguma forma a força final de seus pacientes. Castagna et al., 28 em uma avaliação clínica somente de pacientes submetidos à RTT, descreveram que utilizaram um sistema de teste muscular chamado Lafayette; porém, nos resultados, não especificaram valores, apenas relataram um aumento significativo na pontuação na escala de Constant-Murley, e que todos os parâmetros desta escala melhoraram. Fama et al., 19 por exemplo, obtiveram aumento significativo da força final ao utilizar a RPCL. Eles citam valores médios de força pré-operatórios de 4,4 kg e pós-operatórios de 9,9 kg, mas não especificam se o ombro em questão era o do LD ou o do LND. 19

Entendemos que o acompanhamento dos pacientes por tempo de seguimento maior sempre nos ajuda a entender melhor a evolução no longo prazo. Em comparação com nosso trabalho anterior, 21 quase que dobramos o tempo de seguimento, mas principalmente aumentamos o tempo mínimo de seguimento para 6 anos. Dos poucos trabalhos com seguimento de longo prazo, apresentamos seguimento mínimo maior do que o de Vap et al. 23 Este é o primeiro trabalho publicado até o momento, em língua inglesa, em que se faz uma comparação clínica das técnicas utilizadas discorrendo especificamente sobre a variável força, que inclusive foi estatisticamente significante a favor dos pacientes submetidos a técnica de RTT. Consideramos isto um ponto forte e um fator de importância para a divulgação entre os especialistas.

Como definição de conduta, nosso grupo opta por realizar o reparo das LPPAMR pela técnica de RTT quando se trata de pacientes jovens cuja lesão tenha origem traumática, e naqueles que praticam esportes que requerem força do membro superior, visto ser esta a única variável que obtém melhora estatisticamente significativa. Já nos pacientes idosos cuja lesão tem origem degenerativa e que não necessitam de ganho de força substancial para a prática esportiva, a técnica de RPCL é a escolhida, pela facilidade e tempo cirúrgico diminuído, pois sabemos que esta técnica resultará em melhora significativa da dor e função do ombro.

## Conclusão

Concluímos que o tratamento artroscópico das LPPAMR, tanto pela técnica de RTT quanto pela técnica de RPCL, atingiu uma pontuação média satisfatória na escala da UCLA, sem diferença estatística. Obtivemos também pontuações médias satisfatórias na escala de Constant-Murley para ambas as técnicas, sem diferença estatística entre elas. Tampouco houve diferença estatística entre a primeira e a segunda avaliações considerando a mesma técnica utilizada.

Após 6 anos, os pacientes tratados pela técnica de RTT atingiram estatisticamente força média maior no LD em comparação com o LND e maior do que os pacientes tratados pela técnica de RPCL.
